# Human-Human Interaction Forces and Interlimb Coordination During Side-by-Side Walking With Hand Contact

**DOI:** 10.3389/fphys.2018.00179

**Published:** 2018-03-07

**Authors:** Francesca Sylos-Labini, Andrea d'Avella, Francesco Lacquaniti, Yury Ivanenko

**Affiliations:** ^1^Laboratory of Neuromotor Physiology, Santa Lucia Foundation, Rome, Italy; ^2^Centre of Space Bio-medicine, University of Rome Tor Vergata, Rome, Italy; ^3^Department of Biomedical and Dental Sciences and Morphofunctional Imaging, University of Messina, Messina, Italy; ^4^Department of Systems Medicine, University of Rome Tor Vergata, Rome, Italy

**Keywords:** human gait, interpersonal coordination, interaction forces, arm-leg coordination, EMG activity, locomotor patterns

## Abstract

Handholding can naturally occur between two walkers. When people walk side-by-side, either with or without hand contact, they often synchronize their steps. However, despite the importance of haptic interaction in general and the natural use of hand contact between humans during walking, few studies have investigated forces arising from physical interactions. Eight pairs of adult subjects participated in this study. They walked on side-by-side treadmills at 4 km/h independently and with hand contact. Only hand contact-related sensory information was available for unintentional synchronization, while visual and auditory communication was obstructed. Subjects walked at their natural cadences or following a metronome. Limb kinematics, hand contact 3D interaction forces and EMG activity of 12 upper limb muscles were recorded. Overall, unintentional step frequency locking was observed during about 40% of time in 88% of pairs walking with hand contact. On average, the amplitude of contact arm oscillations decreased while the contralateral (free) arm oscillated in the same way as during normal walking. Interestingly, EMG activity of the shoulder muscles of the contact arm did not decrease, and their synergistic pattern remained similar. The amplitude of interaction forces and of trunk oscillations was similar for synchronized and non-synchronized steps, though the synchronized steps were characterized by significantly more regular orientations of interaction forces. Our results further support the notion that gait synchronization during natural walking is common, and that it may occur through interaction forces. Conservation of the proximal muscle activity of the contact (not oscillating) arm is consistent with neural coupling between cervical and lumbosacral pattern generation circuitries (“quadrupedal” arm-leg coordination) during human gait. Overall, the findings suggest that individuals might integrate force interaction cues to communicate and coordinate steps during walking.

## Introduction

When humans walk side by side they can hold each other hands and cues from interaction force may be advantageous for postural stability (as, for example, in infants and elders or during unstable walking conditions), sport training or physical rehabilitation. For instance, Balash et al. (2007) found that handholding improved gait speed and reduced gait variability in older adults with a high level gait disorder. In addition, people walking side by side unintentionally synchronize their steps, either with or without hand contact (Zivotofsky and Hausdorff, 2007; van Ulzen et al., 2008; Nessler and Gilliland, 2009, 2010; Nessler et al., 2011; Zivotofsky et al., 2012; Roerdink et al., 2017). The unintended interpersonal coordination might involve mirror-neuron networks, since perception of motion of another person (via auditory, visual, haptic or mechanical information) can induce activity in neurons that are also active during the control of movement, action imitation and motor learning (Rizzolatti and Craighero, 2004).

For bimanual and bipedal tasks, an external light contact guidance (haptic tracking) facilitates independent inter-limb coordination (Rosenbaum et al., 2006; Roelofsen et al., 2016), and for manual tracking, haptic/mechanical physical interactions between partners may be mutually beneficial for improving motor performance (van der Wel et al., 2011; Ganesh et al., 2014). For locomotor tasks, some studies have demonstrated that the tactile feedback, when compared to auditory and visual sensory feedback, is the most effective to produce synchrony during overground side by side walking (Zivotofsky and Hausdorff, 2007; Zivotofsky et al., 2012). Furthermore, the feedback provided by enhanced mechanical coupling results in an increase of phase locking during side-by-side treadmill walking (Nessler and Gilliland, 2009; Roerdink et al., 2017). Only few studies investigated forces arising from physical interaction. For example, Sawers et al. (2017) demonstrated that relatively small interaction forces may communicate movement goals during cooperative physical interactions while performing a forward-backward partnered stepping task. Lanini et al. (2017) studied a particular case of interactive locomotion where two humans carried a rigid object and they developed a 2D model, made of two coupled spring-loaded inverted pendulums to reproduce human locomotor behavior. However, despite the importance of haptic interaction in general and the natural use of hand contact between humans during walking, to our knowledge there are no studies that examined and interpreted the interaction forces during side-by-side walking with hand contact to understand the neuro-mechanical processes underlying human-human physical interactions. In addition, such studies may also provide insights into the role of interaction forces in the dyad's ability to communicate and interpret intended motion during locomotion.

The aim of this study was to characterize the interpersonal interaction forces occurring during different interpersonal synchronization modes while walking with hand contact. To isolate the role of haptic interaction, only hand contact-related sensory information was available for unintentional synchronization, while visual and auditory communication was blocked by obstructing peripheral visual feedback of another participant and using headphones that supplied white noise to block out sounds. We also analyzed whole body kinematics and upper limb muscle activity accompanying human-human interaction and changes in the motor patterns during walking with hand contact.

## Methods

### Participants

Sixteen healthy volunteers (mean age 38 ± 8 years [mean ± SD], 8 males and 8 females, mean height 1.78 ± 0.04 m for males and 1.66 ± 0.05 m for females, mean weight 75.5 ± 10.2 kg for males and 55.9 ± 7.1 kg for females) participated in the study and were paired into eight different dyads. The sample size (*n* = 16) was calculated before data collection based on information from previous studies (Nessler and Gilliland, 2010; Lanini et al., 2017). In order to limit the effect of leg length (Nessler and Gilliland, 2009) and sex differences, each subject was assigned to an unique dyad minimizing the height difference between partners and including only subjects of the same sex in the same dyad. The studies conformed to the Declaration of Helsinki, and written informed consent was obtained from all participants according to procedures approved by the Ethics Committee of the Santa Lucia Foundation (protocol CE-PROG.273-22).

### Experimental setup

**Figure 1:**
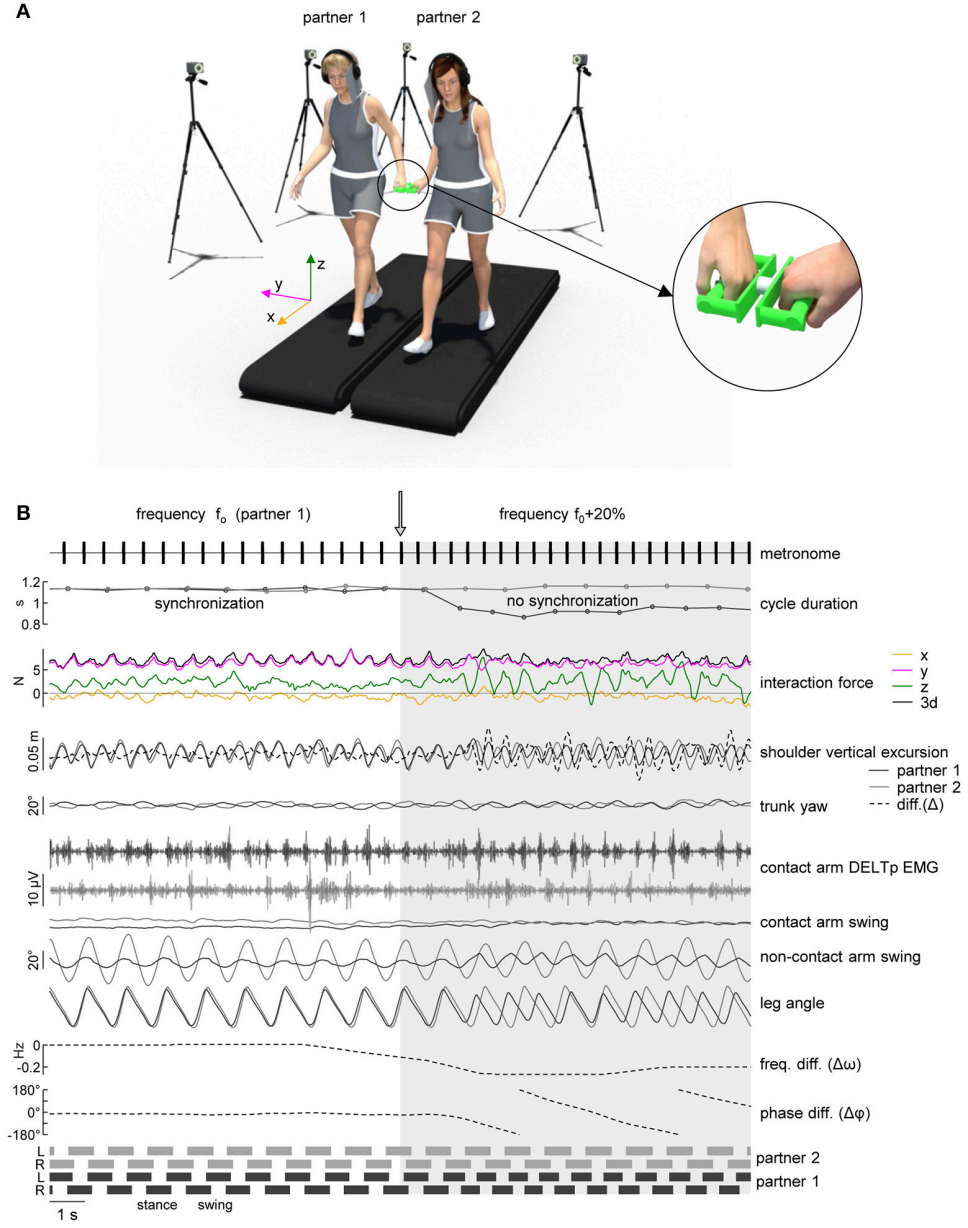
Experimental setup. **(A)** Schematic illustration of the experimental setup and of the force sensing device (*right panel*) with a load cell in the center and handles attached to each side. **(B)** An example of cycle duration, interaction force, kinematic parameters and EMG activity when the metronome pace provided to one of the partners was switched from f_0_ (natural stride frequency) to f_0_+20% (instant denoted by the vertical arrow). *From top to bottom*: metronome audio waveform sent to “partner 1” headphones, gait cycle duration of the contact side leg (left leg for partner 1 in black, right leg for partner 2 in gray), interaction force about x, y, and z axes (see panel **A** for axes orientation) and resultant tridimensional force magnitude (3d), vertical displacement of the contact side shoulder marker [dashed line represents the vertical distance between the shoulder markers of the two partners, diff. (Δ)], yaw angle of the upper trunk, EMG activity of posterior deltoid of the contact arm, contact and non-contact arm swing angle, contact side leg angle, average frequency difference (Δω) and phase difference (Δφ) of the contact side leg movements of the two partners. At the bottom: lower limb stance durations for each partner.

Two treadmills (Enraf-Nonius EN-Tred 1475.911 and EN-Mill 3446.527; Rotterdam, NL) were positioned side by side not being in contact (at a distance of ~85° cm between the centers of the belts, Figure 1A). Subjects walked at 4° km/h wearing shoes or sneakers. A 1–2° min training period of walking was allowed for each subject before the actual data collection was begun, until he/she could walk comfortably, swinging his/her arms naturally. Each of the two participants was randomly assigned to one of the treadmills and used the same treadmill for all the experimental session. Two wireless stereo headphones (Meliconi HP300 professional, Bologna, IT) were used to mask footsteps sound with white noise or to provide auditory pacing signals (44° kHz, 70° ms tone bursts, Osaki et al., 2008). Two light cardboard panels were attached to the headphones on the contact arm side in order to prevent peripheral vision of the walking partner (Figure 1A). Before the beginning of the experimental session, each participant was asked to stand still on the treadmill wearing the headphone emitting white noise and to increase the sound volume until he/she was unable to hear the footsteps sound produced by the other participant walking on the adjacent treadmill at 4° km/h.

Eight different trials were recorded consecutively with a 2° min break between them (Table 1). Participants were instructed either to walk at their natural stride frequency if they heard white noise or to follow the metronome beat. In order to evaluate unintentional coupling, no instructions about interpersonal synchronization were provided to the subjects. Table 1 summarizes all trials performed by the subjects and Table 2 refers to the abbreviations used for the recorded conditions. In trials t1-3, recorded at the beginning of the experimental session, the two partners of the dyad walked simultaneously but independently (having no visual, auditory, haptic or mechanical feedback about the other partner gait) on the two treadmills. After t1 (normal walking trial) the natural stride cadence (f_0_) was calculated for each subject from the kinematic data, while t2 and t3 were used to verify if he/she was able to properly follow the metronome beat when the metronome pace switched from f_0_ (in the first minute of the trial) to f_0_-20% or f_0_+20% (in the second minute of the trial). In trials t4-8, recorded in a random order, each partner was asked to hold one of the handles connected to the force sensor (see *Data recording*, Figure 1A). The duration of trial t4 was 1 min and white noise was provided to the headphones of both subjects. In trials t5-8, one of the partners was provided with the metronome beat at f_0_ during the first minute of the trial that switched to f_0_-20% or f_0_+20% in the second minute (Figure 1B), and the other partner was provided with white noise. During these trials, only hand contact-related sensory information was available for unintentional synchronization.

**Table 1:** Description of trials.

		**1st minute**		**2nd minute**	
**Trial**	**Contact**	**Right partner**	**Left partner**	**Right subject**	**Left subject**
t1	no	wn	wn	–	
t2		f_0_	f_0_	f_0_−20%	f_0_−20%
t3		f_0_	f_0_	f_0_+20%	f_0_+20%
t4	yes	wn	wn	–	
t5		wn	f_0_	wn	f_0_−20%
t6		wn	f_0_	wn	f_0_+20%
t7		f_0_	wn	f_0_−20%	wn
t8		f_0_	wn	f_0_+20%	wn

*wn, white noise; f_0_, metronome at f_0_ frequency*.

**Table 2:** Abbreviations for the conditions of the analyzed subject.

**Abbreviation**	**Contact**	**Audio**		**Trials used for the analyzed subject**	
		**Analyzed subject**	**Other subject**	**Right partner**	**Left partner**
NC_wn	no	wn		t1	t1
NC_f_0_		f_0_		t2^a^,t3^a^	t2^a^,t3^a^
NC_f_0_−20%		f_0_−20%		t2^b^	t2^b^
NC_f_0_+20%		f_0_+20%		t3^b^	t3^b^
C_wn	yes	wn	wn	t4	t4
C_Of_0_	yes	wn	f_0_	t5^a^,t6^a^	t7^a^,t8^a^
C_Of_0_−20%		wn	f_0_−20%	t5^b^	t7^b^
C_Of_0_+20%		wn	f_0_+20%	t6^b^	t8^b^
C_Af_0_	yes	f_0_	wn	t7^a^,t8^a^	t5^a^,t6^a^
C_Af_0_−20%		f_0_−20%	wn	t7^b^	t5^b^
C_Af_0_+20%		f_0_+20%	wn	t8^b^	t6^b^

*NC, no contact; C, contact; O, other subject; A, analyzed subject; wn, white noise; f_0_, metronome at f_0_ frequency; ^a^first minute of the trial, ^b^second minute of the trial*.

### Data recording

We recorded kinematic data bilaterally at 200 Hz by means of 10 Vicon Bonita cameras (Oxford, UK) spaced around the treadmills. Infrared reflective markers (diameter 1.4 cm) were attached on each side of each subject to the skin overlying the following landmarks: the wrist, elbow, gleno-humeral joint (SHO), greater trochanter (GT), lateral femur epicondyle, lateral malleolus (LM), and fifth metatarso-phalangeal joint. In addition, 7 markers were placed on the force recording device in order to track its position and orientation in space.

EMG activity was recorded bilaterally by means of surface electrodes from 5 upper limb muscles simultaneously: anterior deltoid (DELTa), posterior deltoid (DELTp), medial deltoid (DELTm), flexor carpi ulnaris (FCU), extensor carpi ulnaris (ECU)., Two additional muscles were recorded only for the contact arm: long head of biceps brachii (BIC) and long head of triceps brachii (TRIC). The EMG data were recorded with the wireless Delsys Trigno EMG system (Delsys Inc., Boston, MA), bandwidth of 20–450 Hz, overall gain of 1000, and digitized at 1,000 Hz.

Interaction forces between the two partners were recorded using an ATI Nano25 six-axis force/torque sensor (Apex, North Carolina, USA) with two custom-made wood/aluminum handles attached to both sides of the sensor to provide hand contact for the subjects (Figure 1A) (Italian patent 102016000132368). Force data were digitalized at 1,000 Hz. Prior to the experimental session, baseline voltage levels of all three (x,y,z) force components (while the sensor was placed on a surface and oriented horizontally) were recorded and subtracted from the collected data during the subsequent data analysis. Sampling of kinematic, EMG and force data was synchronized.

### Gait kinematics and interpersonal coordination

Gait cycle was defined as the time between two successive foot contacts by the same leg according to the local maxima of the leg (GT-LM) elevation angle (La Scaleia et al., 2014). The timing of the lift-off was determined analogously identifying the local minima of the leg angle. Upper limb abduction (frontal plane) and swing (sagittal plane) angles were calculated respectively from the vector from the shoulder to wrist markers. The kinematic data were low pass filtered at 20 Hz with a zero-lag 4th order Butterworth filter.

In order to evaluate the coupling between the two partners, we applied the method described by van Ulzen et al. (2008) to the contact side leg elevation angles of each partner (Figure 1B). Briefly, the power spectrum of these angles was calculated using a fast Fourier transform over a 5 s window (step size of 1 sample) and leg movement frequency (i.e., stride frequency, ω) was identified as the frequency with the higher peak in the (time-resolved) power spectrum. Frequency locking was present whenever the mean frequency difference (Δω, Figure 1B) between the subjects over the sliding window was smaller than 2 × 10^−4^ Hz (van Ulzen et al., 2008). To obtain time-resolved relative phases between the legs, signals were band-pass filtered (4th order zero-lag Butterworth filter) using an individual frequency band centered around the movement frequency (0.7 × minimum movement frequency, 1.3 × maximum movement frequency). After that, the analytical signal was computed via the Hilbert transform and its phase (φ) was used as instantaneous phase (van Ulzen et al., 2008). Using the sliding window method, synchronized moments were defined as those that fulfilled the following two criteria: (1) frequency locking was present, and (2) the phase difference (Δφ, Figure 1B) between the two partners had a trend (calculated from linear regression of Δφ over the sliding window) smaller than 5 s. Because of the 5 s sliding window, 2.5 s were excluded from the beginning and from the end of each time-series, and also the 5 s in the middle of the 2 min trials were excluded to avoid transients (Table 1).

### EMG data analysis

EMG data were processed using standard filtering and rectifying methods. The raw EMG signals were high-pass filtered at 30 Hz, full-wave rectified, and low-pass-filtered at 10 Hz (all filters, zero-lag 4th order Butterworth). Kinematic and EMG data were time-interpolated over individual gait cycles to fit a normalized 200-point time base and averaged across cycles and then across subjects. In addition, we calculated for each muscle and each subject the center-of-activity (COA) throughout the gait cycle. The COA during the gait cycle was calculated using circular statistics (“circ_mean.m” function in the CircStat Matlab toolbox, Berens, 2009) and plotted with polar plots: polar direction denoted the phase of the movement cycle (with angle θ that varies from 0 to 360° corresponding to 0 and 100% cycle). The COA of the EMG waveform was calculated as the angle of the vector (first trigonometric moment), which points to the center of mass of that circular distribution using the following formulas:

(1) $$\begin{array}{r} A=\sum_{t=1}^{200}\left(\cos\theta_{t}\times EMG_{t}\right) \end{array}$$

(2) $$\begin{array}{r} B=\sum_{t=1}^{200}\left(\sin\theta_{t}\times EMG_{t}\right) \end{array}$$

(3) $$\begin{array}{r} COA=\tan^{-1}\left(B/A\right) \end{array}$$

The COA has been previously used to characterize the overall temporal shifts of EMG or motoneuron activity (Yakovenko et al., 2002; Ivanenko et al., 2006a; Sylos-Labini et al., 2011, 2014b) and was chosen because it was impractical to reliably identify a single peak of activity in the majority of muscles. It can be helpful for evaluating if the distribution of muscular activity remains unaltered across different conditions.

In addition, we extracted the basic activation patterns from the EMG activity of 6 bilateral deltoid muscles (most active during walking, see Results) for each participant. We applied a non-negative matrix factorization (NMF) of the EMG data of individual gait cycles (low pass filtered at 5 Hz, the minimum over the cycle was subtracted from each profile) using the algorithm described by Lee and Seung (1999) that constrains the basic patterns and weights to be non-negative. To determine the number of significant basic patterns, we performed an iterative reconstruction of the EMG profiles from individual participants using *k* = 1–8 patterns, until the variance accounted for (VAF) by these patterns was greater or equal 80%, that is, the residual error accounted for less than 20% of data variance (Ivanenko et al., 2004, 2005; Clark et al., 2010).

To identify and average similar basic activation patterns across cycles, all activation patterns extracted from all cycles were partitioned into *n* mutually exclusive patterns, for each subject and each condition, using the k-means (Matlab function kmeans.m) clustering algorithm (Hartigan and Wong, 1979; Saltiel et al., 2001; Kim et al., 2016). Briefly, k-means treats each observation in the data as an object having a location in space. It finds a partition in which objects within each cluster are as close to each other as possible, and as far from objects in other clusters as possible. Each cluster in the partition is defined by its member objects and by its centroid, or center. The centroid for each cluster is the point to which the sum of distances from all objects in that cluster is minimized. The distance measure, in 200-dimensional space, used for minimization was one minus the cosine of the included angle between points (treated as vectors). To evaluate the optimal number of clusters m, we utilized the average silhouette value. The silhouette value is a measure of how similar that point is to points in its own cluster, when compared to points in other clusters. The silhouette value for the i-th point, S_i_, is defined as

(4) $$\begin{array}{r} S_{i}=\frac{\left(b_{i}-a_{i}\right)}{\max\left(a_{i},b_{i}\right)} \end{array}$$

where *a*_*i*_ is the average distance from the i-th point to the other points in the same cluster as *i*, and *b*_*i*_ is the minimum average distance from the i-th point to points in a different cluster, minimized over clusters. The silhouette value ranges from −1 to +1. A high silhouette value indicates that i is well-matched to its own cluster, and poorly-matched to neighboring clusters. If most points have a high silhouette value, then the clustering solution is appropriate. The basic patterns with S < 0.2 where considered unmatched and excluded from the cluster. For each group, the resulting clusters of basic patterns were plotted in a chronological order (i.e., according to the timing of the main peak relative to the gait cycle). To identify and average similar basic activation patterns across subjects for each condition, the centroids of the clusters of all subjects were clustered using the same procedure described above.

### Interaction forces

The tridimensional forces were transformed from sensor to fixed coordinate system (x: direction of locomotion, y: lateral direction, z: vertical direction, Figure 1A). Force data were low-pass-filtered at 20 Hz with a zero-lag 4th order Butterworth filter. The time course of each force component and of the resultant 3d force was expanded in Fourier series, and the percent variance accounted for by the 1st and 2nd harmonics was calculated.

In addition to analyzing the amplitude and time-series of interaction forces, we also examined the orientation of interaction forces in space and its variability. A similar method has been used for characterizing the orientation of the covariation plane of the limb segment elevation angles (Sylos-Labini et al., 2017). To assess variability, the spherical contour of the density distribution of the tridimensional force vector was calculated over 5 consecutive strides adapting in Matlab the algorithm proposed by Vollmer (1995) and based on the modified Kamb method (Kamb, 1959). Briefly, if *n* points are selected randomly from a uniform population distributed over an area *A*, the probability that any given point will lie within an arbitrary subarea *a* of *A* is *p* = *a*/*A*. The number of points occurring within area *a* can be considered as a binomial random variable (which mean is μ = *np* and standard deviation is $\sigma =\sqrt{np\cdot \left(1-p\right)}$) with an expected count *E*, equal to the mean μ. Kamb (1959) selected a binomial probability model with *E* = μ = 3σ so that, given a random sample from a uniform population, the counting circle would be large enough so the observed counts would not be likely to fluctuate wildly from the expected count. Contour levels greater than 3σ(*E*) indicate a density higher than expected for a uniform distribution, and levels less than 3σ indicate a density lower than expected. The 5σ contour, for example, represents densities 2SDs larger than expected (*E*+2σ). In the algorithm, the nodes of a regular square (30 × 30) grid are back-projected onto the sphere using a stereographic projection. For each node on the sphere, the number of data points that fall within a spherical cap of area *a* = 2*π* · (1 − cos *θ*) (where θ is the semi-apical angle of the cap) were counted with an exponential weighting function in order to smooth the contour. For directed data distributed on a unit hemisphere of area 2π, the angle θ can be calculated considering that *p* = *a*/*A* = (1 − cosθ)/2. The contour levels were drawn using linear interpolation through the grid (Vollmer, 1995) and the area of the *E* + 2σ (= 5σ) contour and the maximum density level were used as a measure of spatial variability.

### Statistics

For the analysis of interpersonal coordination, kinematic and EMG data, we considered each single subject independently (n = 16). The data acquired for each trial were assigned to 11 conditions (Table 2) based on the combination of the following experimental features: the presence of hand contact (no or yes), the auditory signal sent to the subject (white noise or metronome at f_0_, f_0_–20% or f_0_+20%) and the auditory signal sent to his/her partner in the dyad. Consequently, the data acquired from 2 min trials were divided into two separate time-series lasting 1 min according to the condition (Table 1). Mean values were calculated over all strides (of the contact side leg) for each condition and subject. Overall means and standard deviations were computed across subjects. In order to assess differences between the means of the conditions described in Table 2, we used repeated-measures analysis of variance (RM-ANOVA) and post-hoc Dunnett's test to evaluate the differences with the normal walking condition (NC_wn). Circular statistics on directional data (Batschelet, 1981; Fisher, 1995) were used to characterize the mean COA for each muscle (see preceding text) and its variability across subjects (angular SD). The Watson-William test was used for COA to evaluate the difference between walking with or without hand contact (conditions C_wn and NC_wn).

We also analyzed the relationship between different modes of interpersonal gait coordination (stride synchronization and/or phase shift between leg movements of two partners) and interaction forces (or kinematic data). To this end, for each dyad, we selected intervals of 5 consecutive strides (~5 s)—denoted by similar features of the locomotor coupling—representing four different modes of interpersonal coordination. The first three modes were identified by synchronized strides (|Δω| < 0.0002 Hz) and separated according to the phase shift between the contact side leg movements (interval between the heel strikes in %cycle) of the two partners: (1) in-phase (Δφ < 5% or Δφ > 95%), (2) anti-phase (45% < Δφ < 55%) or (3) out-of-phase (10% < Δφ < 40% or 60% < Δφ < 90%) coordination. The fourth (non-synchronized) mode included only the intervals characterized by strong detuning (|Δω|>0.18 Hz) to emphasize the differences between synchronized and not-synchronized walking. Overall means and standard deviations of all parameters were computed over the 5-strides intervals. We used one-way ANOVA to evaluate differences between the four modes of interpersonal coordination and RM-ANOVA to evaluate the differences within the percent variance accounted for by the 1st and 2nd harmonics between the four modes of interpersonal coordination. We performed the multiple comparison of the means using the post-hoc Fisher's least significant difference (LSD) test. Least-squares polynomial fitting and Pearson correlation coefficients were used to analyze the relationship between interaction forces and interpersonal gait parameters (interpersonal gait synchronization, phase shift, inter-subject shoulder distance). Reported results are considered significant for *p* < 0.05.

## Results

### Stepping frequency

**Figure 2:**
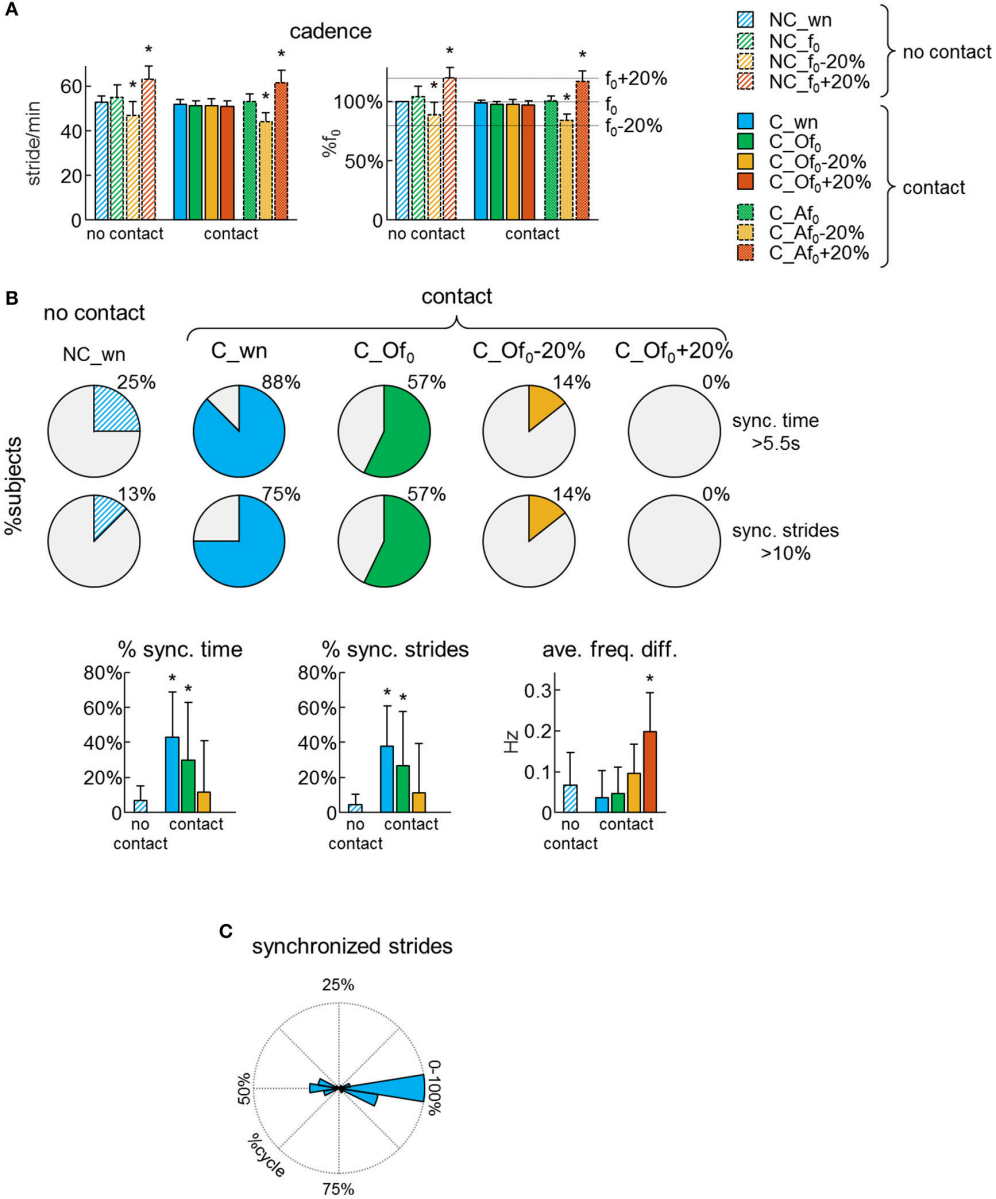
Dyad's synchronization. **(A)** Average (+SD) cadence expressed in stride/min (left panel) and as a percentage of the natural stride frequency (f_0_) of each subject (right panel) across different conditions (see Table 2 for the abbreviations). **(B)** Pie charts illustrating, for each condition, the percentage of subjects (*n* = 16), which experienced synchronized gait with the partner for at least 5.5 s (upper pies) or at least 10% of strides (lower pies). *At the bottom*: averaged (+SD) percent of time of synchronized (sync.) locomotion (left), percent of synchronized strides (middle) and average frequency difference of the contact side leg movements of the two partners (right) across different conditions. Asterisks denote significant (Dunnett's *post-hoc* test *p* < 0.05) differences with the normal (no contact) walking condition (NC_wn). **(C)** Polar histogram illustrating the distribution of the phase shift between contact side leg movements (Δφ) for the synchronized strides.

Participants were asked to walk on a treadmill at 4 km/h, either at their natural walking cadence (f_0_) or following the beat of a metronome at f_0_, f_0_−20% or f_0_+20%. Each subject was able to adapt his/her cadence to the metronome beat during walking without or with hand contact. Accordingly, the average cadence was significantly different from f_0_ when the subjects were asked to walk at a pace that was 20% higher or lower than f_0_ [RM-ANOVA *F*_(10, 150)_ = 54.3 *p* < 0.001, Dunnett's test *p* < 0.001 in conditions NC_f_0_−20%, NC_f_0_+20%, C_Af_0_−20%, and C_Af_0_+20%, Figure 2A]. In contrast, when the participants were not hearing any pace cue while the other partner walked at f_0_−20% or f_0_+20%, on average they did not change their natural cadence (Dunnett's test *p* ≥ 0.35 in conditions C_Of_0_−20% and C_Of_0_+20%, Figure 2A).

### Unintended synchronization

Synchronization of steps was assessed using the frequency analysis of leg movements. When the partners walked without hand contact, there was no sensory information available for unintentional step frequency locking, thus we can assume that the low percentage of synchronized strides occurred by chance (Figure 2B). However, when the partners walked with hand contact at natural cadence, 88% of them experienced at least 5.5 s (10% of trial duration) of synchronous steps and the percentage of synchronized time and strides was significantly higher (~40%) than when they walked with no hand contact [RM-ANOVA *F*_(3, 39)_ = 4.35 *p* = 0.01, Dunnett's test *p* ≤ 0.04 for C_wn and C_Of_0_ conditions, Figure 2B lower panels]. Yet, when one of the partners was asked to walk following the metronome with a rhythm f_0_−20% or f_0_+20%, there was no interpersonal synchronization despite the hand contact (Figure 2B).

We also analyzed the phase of leg movements of the partners. For non-frequency-locked strides, the phase difference between the contact side legs wrapped continuously between 0 and 100% of gait cycle. However, for frequency locked strides, it was centered around 0 or 50% of gait cycle (Figure 2C). In particular, 59% of the synchronized strides exhibited an in-phase coordination of the contact side legs (i.e., the left leg of one of the partner moved in phase with the right leg of the other partner), 26% exhibited an anti-phase coordination and only 15% of the synchronized strides presented an out-of-phase coordination (Figure 2C). These findings suggest that some modes of interpersonal coordination are more stable than others, as in the case of inter-limb coordination patterns during bimanual or bipedal tasks (Kelso, 1984; Roelofsen et al., 2016).

### Gait kinematics and EMG patterns of the upper limb muscles

**Figure 3:**
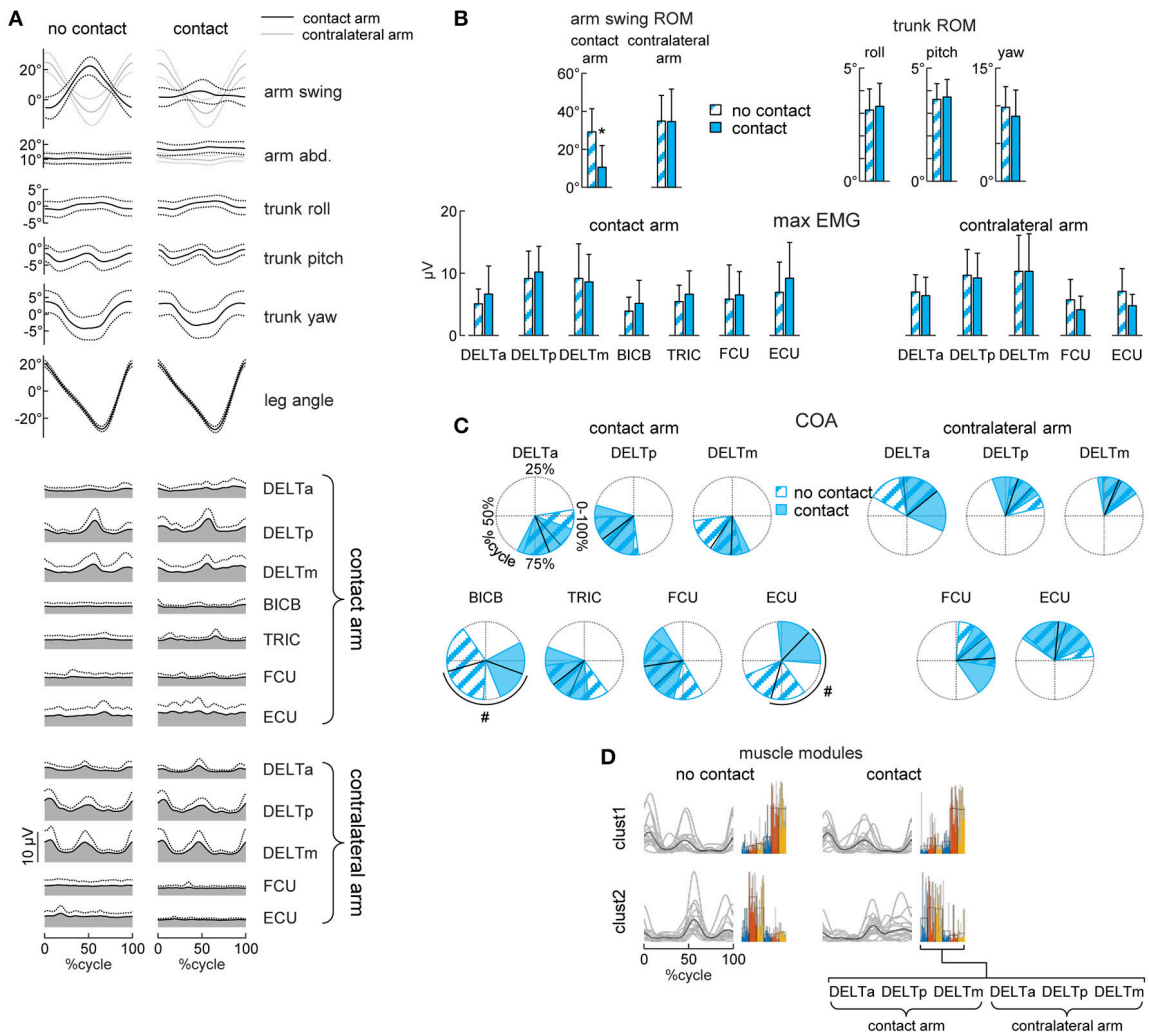
Gait kinematics and upper limb EMG patterns. **(A)** Ensemble averaged (mean ± SD, across subjects) kinematic and EMG patterns during walking without (left column) or with (right column) hand contact. From top to bottom: arm swing and abduction angles (black curves refer to the contact arm while light gray curves refer to the contralateral arm), roll, pitch and yaw angles of the trunk, contact side leg angle, EMG patterns of 7 muscles of the contact arm and 5 muscles of the contralateral arm. Patterns are plotted versus normalized gait cycle calculated from the contact side leg. **(B)** Averaged (+SD) range of motion (ROM) of the arm swing angle, ROM of the roll, pitch and yaw angles of the trunk, maximum EMG activity in the muscles of the contact arm and of the contralateral arm during walking without or with hand contact. **(C)** Polar plots of the center of activity (COA). Polar direction denotes the relative time of the averaged (across subjects) COA over the gait cycle (time progresses clockwise), the width of the sector denotes angular SD. **(D)** Basic muscle activation patterns and their clustering across different conditions. Basic patterns are plotted in a chronological order (with respect to the time of the main peak of the centroid of each cluster–displayed with a black curve). Each gray curve represents the centroid of the clusterization of the basic activation patterns for each subject. By the side of each cluster of patterns, the individual (color bars) and mean (black contour bars) muscle synergies are displayed. Asterisks **(B)** denote significant (Dunnett's *post-hoc* test *p* < 0.05) differences relative to the normal (no contact) walking condition. Hash tags **(C)** denote significant (Watson-Williams test *p* < 0.05) differences relative to the normal walking condition.

The upper body kinematics was assessed by analyzing arm and trunk oscillations. On average, the amplitude of the contact arm swing movements decreased [RM-ANOVA *F*_(10, 150)_ = 26.2 *p* < 0.001, Dunnett's test *p* < 0.001, Figures 3A,B] while the contralateral (free) arm oscillated in the same way as during normal walking at 4 km/h [RM-ANOVA *F*_(10, 150)_ = 3.07 *p* = 0.001, Dunnett's test *p* = 1]. Furthermore, the kinematics of the trunk, as assessed by roll, pitch and yaw angle oscillations, was not influenced by the hand contact (RM-ANOVA or Dunnett's test *p* > 0.05, Figures 3A,B).

Walking with hand contact (holding a handle) could be expected to modify the pattern of activity of the upper limb muscles. To examine this, we analyzed EMG activity of both the contact and contralateral arms and compared it with that during normal walking. During normal walking, the activity of the proximal (deltoid) muscles showed higher amplitude modulating, while that of the distal muscles was generally smaller and more variable (Figure 3A, left column), consistent with previous studies (Ballesteros et al., 1965; Hogue, 1969; Ivanenko et al., 2006b; Kuhtz-Buschbeck and Jing, 2012). During walking with hand contact, there was a significant shift of the center of activity of BICB and ECU muscles of the contact arm (Watson-William test *p* = 0.001 and *p* < 0.001, respectively, Figures 3A,C), likely because holding the handle required some additional activity of distal arm muscles. Nevertheless, their activity was generally small and variable (Figures 3A,B).

Interestingly, despite the substantial reduction of contact arm swing movements, the EMG activity of the shoulder muscles of the contact arm did not decrease (RM-ANOVA or Dunnett's test *p* > 0.05, Figures 3A,B). Moreover, the waveforms remained similar and the center of activity of these muscles did not change during walking with hand contact (Watson-William test *p* > 0.05, Figures 3A,C). To verify further whether the spatiotemporal structure of the shoulder muscle activity (associated with the coupling of the cervical and lumbosacral central pattern generators during human locomotion, Dietz, 2002; La Scaleia et al., 2014; Sylos-Labini et al., 2014a) remained similar, we applied non-negative matrix factorization and cluster analysis. The average number of modules needed to account for at least 80% of variance of the bilateral EMG activity of the six shoulder muscles was similar (paired *t*-test *p* = 0.26) between normal walking (2.2 ± 0.4) and walking with hand contact (2.4 ± 0.5). Furthermore, we found two clusters of basic activation patterns of all subjects both when walking without and with hand contact (Figure 3D). Thus, the hand contact did not significantly change the spatiotemporal structure of the proximal arm muscles (most active during walking), nor the amplitude of trunk oscillations despite considerable attenuation of contact arm swing movements (Figure 3). While Figures 3A,B shows a comparison between normal walking and walking with hand contact without any auditory pace cues (NC_wn vs. C_wn), the hand contact did not affect trunk oscillation and upper limb EMG activity also during other conditions, in which the subjects walked at metronome-imposed cadences (f_0_, f_0_+20%, f_0_−20%, see Supplementary Figures 1, 2).

### Interpersonal interaction forces during walking with hand contact

**Figure 4:**
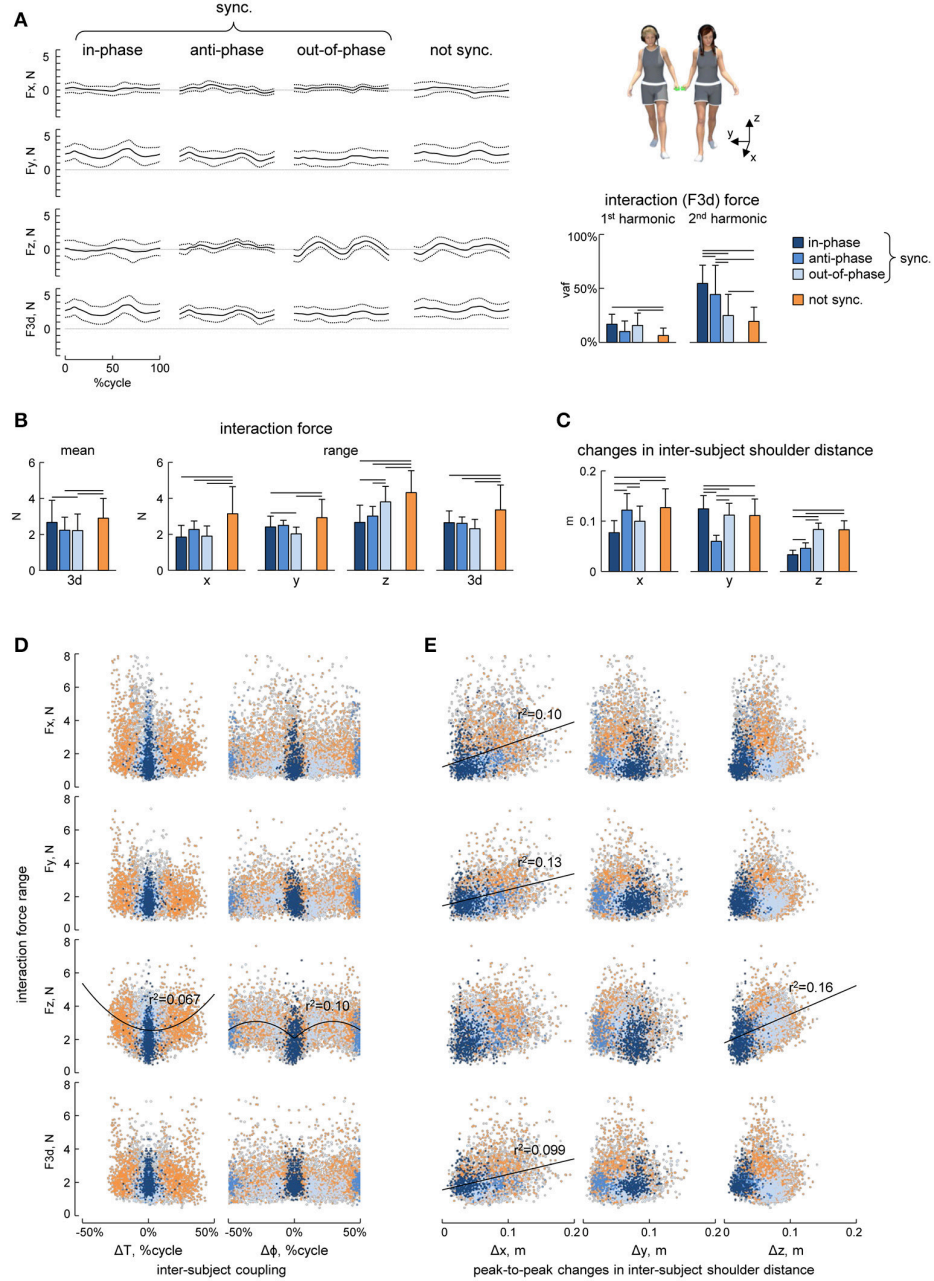
Interaction forces. **(A)** Ensemble averaged (mean ± SD, across strides) interaction force components about x, y, and z axes (F_x_, F_y_, and F_z_) and tridimensional force magnitude (F_3d_, *at the bottom*) across the different modes of interpersonal coordination. *From left to right*: in-phase (Δφ <5% or Δφ >95%), anti-phase (45% < Δφ <55%) and out-of-phase (10% < Δφ <40% or 60% < Δφ <90%) synchronized (|Δω| < 0.0002 Hz) strides and not-synchronized (|Δω|>0.18 Hz) strides. The bar plots *on the right* show the averaged (+SD) percent of variance accounted for (vaf) by the 1st and the 2nd harmonics of the 3d interaction force. **(B)** Averaged (+SD) mean (over the 5 consecutive strides) interaction force about x, y, and z axes and its 3d magnitude (left panel) and the range of changes of the interaction force (right panels). **(C)** Peak-to-peak changes (mean + SD) in the inter-subject shoulder distance along the x, y, and z axes. **(D)** Relationship between interaction force oscillations (about x, y, and z axes and 3d) and interpersonal gait parameters (the pace difference ΔT, calculated as the difference between the durations of two concurrent strides of the partners, left column, and the phase shift Δφ between two strides [heel strikes] of the partners, right column). Each point represents a stride, the color of the points has the same denotation used for the different modes of interpersonal gait synchronization (see the legend in **A**) while light-gray describes points that fall outside of this classification. **(E)** Relationship between interaction force oscillations and the amplitude of changes in the inter-subject shoulder distance along the x, y, and z axes (Δx, Δy, and Δz columns from left to right) in the same format as in panel **D**. Horizontal lines in **A–C** denote significant (LSD *post-hoc* test *p* < 0.05) differences. Black lines in **D,E** indicate power function and linear fitting, respectively, that correlated (*r*^2^ > 0.05) with the data.

Walking side-by-side with hand contact (Figure 1A) provides force interaction cues that may affect gait synchronization (Figure 2). We recorded and analyzed the interaction forces between the partners and their differences across the four different modes of interpersonal gait coordination, namely: in-phase, anti-phase, out-of-phase and lack of synchronization (Figure 4).

In general, the resultant (F_3d_) interaction force was relatively small (~2–3 N, Figure 4B left panel) and the ensemble averaged waveforms of F over the gait cycle displayed some similarities across different modes of interpersonal coordination (Figure 4A). For instance, the mean level of the F_y_ component was higher (due to arm abduction) and the total interaction force (F_3d_) showed two main peaks over the gait cycle for all conditions (Figure 4A). Indeed, the percent of variance accounted for by the 2nd harmonics was significantly higher than that of the 1st harmonics [RM-ANOVA *F*_(1, 266)_ = 179.3 *p* < 0. 001, LSD test *p* < 0.001, Figure 4A right panel]. Nevertheless, there were also some differences across conditions. For example, the percent of variance accounted for by the 2nd harmonics was significantly higher for the in-phase and anti-phase synchronized strides [*F*_(3, 266)_ = 92.6 *p* < 0. 001, LSD test *p* < 0.001, Figure 4A], suggesting more regular force oscillations over the gait cycle. The peak-to-peak amplitude of force oscillations was ~2–4 N during different synchronization episodes (Figure 4B), but it was significantly larger for non-synchronized strides [ANOVA *F*_(3, 266)_ = 14.0 *p* < 0.001, LSD test *p* < 0.018].

**Figure 5:**
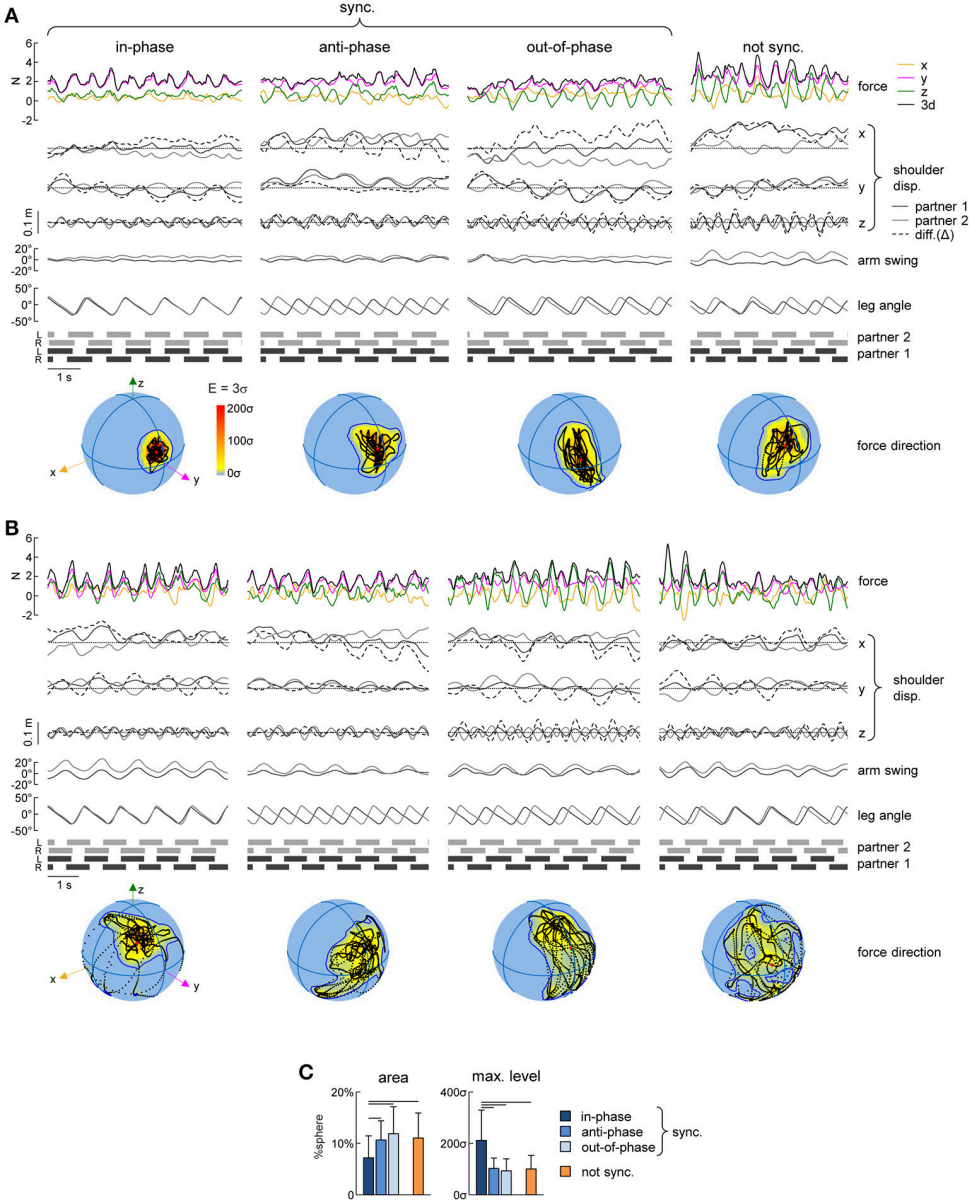
Variability of spatial orientation of interaction forces. **(A)** Examples of interaction forces and arm/leg kinematics of a representative dyad for intervals of 5 consecutive strides during different modes of interpersonal gait synchronization (from left to right): in-phase (Δφ <5% or Δφ >95%), anti-phase (45% < Δφ <55%) and out-of-phase (10% < Δφ <40% or 60% < Δφ <90%) synchronized (|Δω| < 0.0002 Hz) strides and not-synchronized (|Δω|>0.18 Hz) strides. From top to bottom: interaction forces, displacement (x, y, and z) of the contact side shoulder marker (for partner 1 in black, for partner 2 in gray, dashed line represents the difference (Δ) between the partners), yaw angle of the upper trunk, contact arm swing angle, contact side leg angle and lower limbs' stance durations for each partner. At the bottom: Spherical spatial density of the force vector directions during the corresponding 5 consecutive strides. Each point corresponds to a single sample (sample frequency 200 Hz), the color scale indicates density diagrams calculated using the Kamb method for directional data with *E* = 3σ and exponential smoothing (see Methods), and the black contours outline the areas with density equal to *E*+2σ. **(B)** Another example of interactions forces for another dyad (same format as in **A**). **(C)** Averaged (+SD) area of the spherical density ≥ *E*+2σ (left) and maximum level of the spherical density (right) during the different modes of interpersonal coordination. Horizontal lines denote significant (LSD *post-hoc* test *p* < 0.05) differences.

The distance between the subjects was calculated as the distance between the SHO markers of the contact side of the two partners and it oscillated over the gait cycle across all conditions and along all axes (x, y, and z, Figure 4C), which in turn might be related in the amplitude of interaction force oscillations. The range of changes in the inter-subject shoulder distance along the vertical direction (Δz) was significantly smaller for in-phase and anti-phase synchronized strides [ANOVA *F*_(3, 266)_ = 126.8 *p* < 0.001, LSD test *p* < 0.001], since the vertical two-peaked trunk oscillations (due to the pendulum mechanism of walking, (Cavagna and Margaria, 1966; Full and Koditschek, 1999) tended to be synchronous for these conditions (Figures 5A,B). These changes in Δz could likely explain that peak-to-peak oscillations in F_z_ were significantly larger during the out-of-phase synchronization mode and for not-synchronized strides [ANOVA *F*_(3, 266)_ = 26.9 *p* < 0.001, LSD test *p* < 0.018, Figures 4B,D] and that peak-to-peak oscillations in F_z_ and the vertical inter-subject shoulder distance (Δz) correlated (Figure 4E). As for other spatial components, oscillations in F_x_, F_y_, and F_3d_ correlated with peak-to-peak oscillations in the inter-subject shoulder distance (Δx) along the horizontal axis (Figure 4E).

Finally, we also analyzed the orientation of the interaction force in space and its variability using the spherical contours of the density distribution of the F_3d_ vector (see Methods). Figures 5A,B shows some examples of this analysis in two representative dyads during different modes of coordination. Changes in the direction of the interaction force over gait cycles corresponded to black points on the spheres in the examples shown in Figures 5A,B and they tended to be concentrated over a smaller area of the sphere (indicated by the contour corresponding to the average density +2SD) in the case of in-phase synchronization. In fact, for all dyads, the area of the E+2σ spherical contour was significantly smaller for the in-phase synchronized strides than in all other conditions [*F*_(3, 266)_ = 8.05 *p* < 0.001, LSD test *p* < 0.023] (Figure 5C, left panel). This result is reinforced by the fact that also the maximum density of the spherical density plots was on average two times greater for the in-phase synchronized strides compared to the others [*F*_(3, 266)_ = 33.2 *p* < 0.001, LSD test p < 0.001] (Figure 5C, right panel).

## Discussion

In this study, we described a novel approach to evaluate human-human interaction forces during side-by-side walking with hand contact. The results (Figure 2) are consistent with the previous studies indicating that, when walking hand-in-hand, people tend to synchronize their strides even if they have no auditory or visual information about the movements of their partners (van Ulzen et al., 2008; Nessler et al., 2011; Zivotofsky et al., 2012; Roerdink et al., 2017). We also found that, even though the participants reduced oscillations of the contact arm, the rhythmic patterns of EMG activity of the proximal upper limb muscles were similar to those of normal walking when both upper limbs were oscillating (Figure 3). Finally, we analyzed the spatiotemporal characteristics of the interaction forces between the subjects' hands associated with different modes of interpersonal synchronization (Figures 4, 5). Below we discuss the results in the context of interlimb coordination and human-human interaction during walking with hand contact.

When the partners walked with hand contact, a substantial reduction in the contact arm swinging occurred as if the subjects tried to stabilize the contact point, while the contralateral (free) arm continued oscillating normally (Figures 3A,B). This reduction was observed in all conditions, including both synchronized and non-synchronized strides (see Supplementary Figure 1). Interestingly, despite the significant reduction of limb movement, we found that the EMG activity of shoulder muscles did not decrease, as well as their basic activation patterns (using non-negative matrix factorization and cluster analysis) remained similar to those obtained during normal walking (Figures 3A,C,D). These results are reminiscent to the findings of Kuhtz-Buschbeck and Jing (2012) that showed phasic activity of arm and shoulder muscles also when arm swing was deliberately avoided during walking. Furthermore, trunk oscillations (including trunk torsion) were not influenced by hand contact (Figures 3A,B, see also Supplementary Figure 1). The conservation of the proximal upper limb muscle activity of the contact (not oscillating) arm supports the idea of neural coupling between cervical and lumbosacral pattern generation circuitries (“quadrupedal” arm-trunk-leg coordination) during human gait (Dietz, 2002; Zehr and Duysens, 2004; de Sèze et al., 2008; Meyns et al., 2013; La Scaleia et al., 2014; Sylos-Labini et al., 2014a).

Step synchronization frequently occurs when people walk side-by-side either with or without hand contact and the tactile feedback, when compared to auditory and visual sensory feedback, seems to be the most effective to produce synchrony (Zivotofsky and Hausdorff, 2007; Zivotofsky et al., 2012). Our results further support the notion that gait synchronization during natural walking is common and may occur through interaction forces when two individuals are in hand contact and audiovisual feedback is not available, yet we found that this phenomenon does not normally occur when the cadence difference is about 20% (Figure 2). Moreover, our findings suggest that some modes of interpersonal coordination are more frequent than the others (Figure 2C), consistent also with the existence of more stable and less stable patterns during bimanual or bipedal inter-limb coordination (Kelso, 1984; Roelofsen et al., 2016).

The occurrence of different synchronization modes was previously reported for locomotion. Similarly to the results of Nessler and Gilliland (2009) about enhanced mechanical coupling, we found that the occurrence of in-phase coordination of the contact side legs was almost twice more frequent than that of anti-phase during walking with hand contact (Figure 2). On the other hand, Zivotofsky and Hausdorff (2007) found that both in-phase and anti-phase coordination are similarly frequent during overground locomotion, while Roerdink et al. (2017) reported that ipsilateral phase-locking prevailed over contralateral phase-locking during walking hand-in-hand at 1.3 km/h. Such discrepancies may be related to some differences between treadmill vs. overground walking (e.g., it reduces the natural variability of speed, stride length, and stride time, Frenkel-Toledo et al., 2005) or very slow walking velocity (1.3 km/h). For instance, walking speed affects the kinematics and relative phase dynamics of trunk oscillations (van Emmerik and Wagenaar, 1996) and, consequently, it could affect the human-human coordination when walking with hand contact. Nevertheless, whatever the exact percentage of different modes of synchronization under different walking speed conditions, our results showed that they may be associated with different characteristics of interactions forces (Figures 4,5).

To quantify the interpersonal interaction forces, we analyzed both the contribution of different force components (x,y,z) and the spatiotemporal characteristics of the amplitude and orientation of the resultant interaction force. To our knowledge, our study represents the first attempt to evaluate the interaction forces that occur during side-by-side walking with hand contact. Several features of dynamic haptic interactions can be noted (Figures 4,5). First, the amplitude of the interaction force (~3 N) was comparable or smaller than the forces measured during other human-human interaction tasks (Ikeura and Inooka, 1995; Reed et al., 2005; Reed and Peshkin, 2008; Wang et al., 2008; Hawkins et al., 2012). For example, the human-human interaction forces reported during a forward-backward partnered stepping task were ~5–12 N, consistent with the idea that small forces are effective for sensorimotor human-human communication (Sawers et al., 2017). Second, as far as it concerns different force components, the mean level of the lateral (y) component was larger (due to arm abduction) (Figure 4A) while the amplitude of oscillations was roughly comparable for the three force components (Figure 4B). Finally, in addition to some differences in the force magnitude across different types of interpersonal synchronization (Figure 4), systematic changes in the variability of force orientation (Figure 5) suggest that the latter information may be essential for haptic communication.

In conclusion, the findings suggest that individuals might integrate force interaction cues to communicate and synchronize steps or optimize coordination during walking. Side-by-side walking with hand contact is a common situation that we naturally experience since infancy. The relationship between force and motion represents an important means for communicating between biological agents (Rosenbaum et al., 2006; van der Wel et al., 2011; Ganesh et al., 2014; Roelofsen et al., 2016; Lanini et al., 2017; Mojtahedi et al., 2017; Sawers et al., 2017). The developed approach and metrics to assess the sensory and motor mechanisms underlying human-human interaction, and the directional forces and their variability in particular, can be used to successfully identify interactions during various locomotor tasks, such as haptic guidance during dimensional locomotion, unstable walking, etc. Investigating the basic principles that drive human-human haptic interaction during walking may also be important for understanding the sensory and neural processes underlying locomotor learning, sport training, gait rehabilitation, interaction between agents of different size (e.g., child-adult), as well as human-robot interactions in motor assistive tasks.

## Author contributions

Conceived and designed the experiments: FS-L, AdA, FL, and YI; Performed the experiments and analyzed the data: FS-L and YI. All the authors made contributions in writing the manuscript and interpreting the results and have approved the final version.

### Conflict of interest statement

The authors declare that the research was conducted in the absence of any commercial or financial relationships that could be construed as a potential conflict of interest.
