# Supplementary material for: Human-Human Interaction Forces and Interlimb Coordination During Side-by-Side Walking With Hand Contact
Source: Front Physiol. 2018 Mar 7;9:179. doi: 10.3389/fphys.2018.00179 (PMC5850283; doi:10.3389/fphys.2018.00179)
Supplement: Supplementary file 1 [file Image1.PDF]

Supplementary Material

Human-human interaction forces and interlimb coordination during side-by-side walking with hand contact

Francesca Sylos-Labini1*, Andrea d'Avella, Francesco Lacquaniti, Yury Ivanenko

*** Correspondence:** Francesca Sylos-Labini: f.syloslabini@hsantalucia.it

1 Supplementary Figures

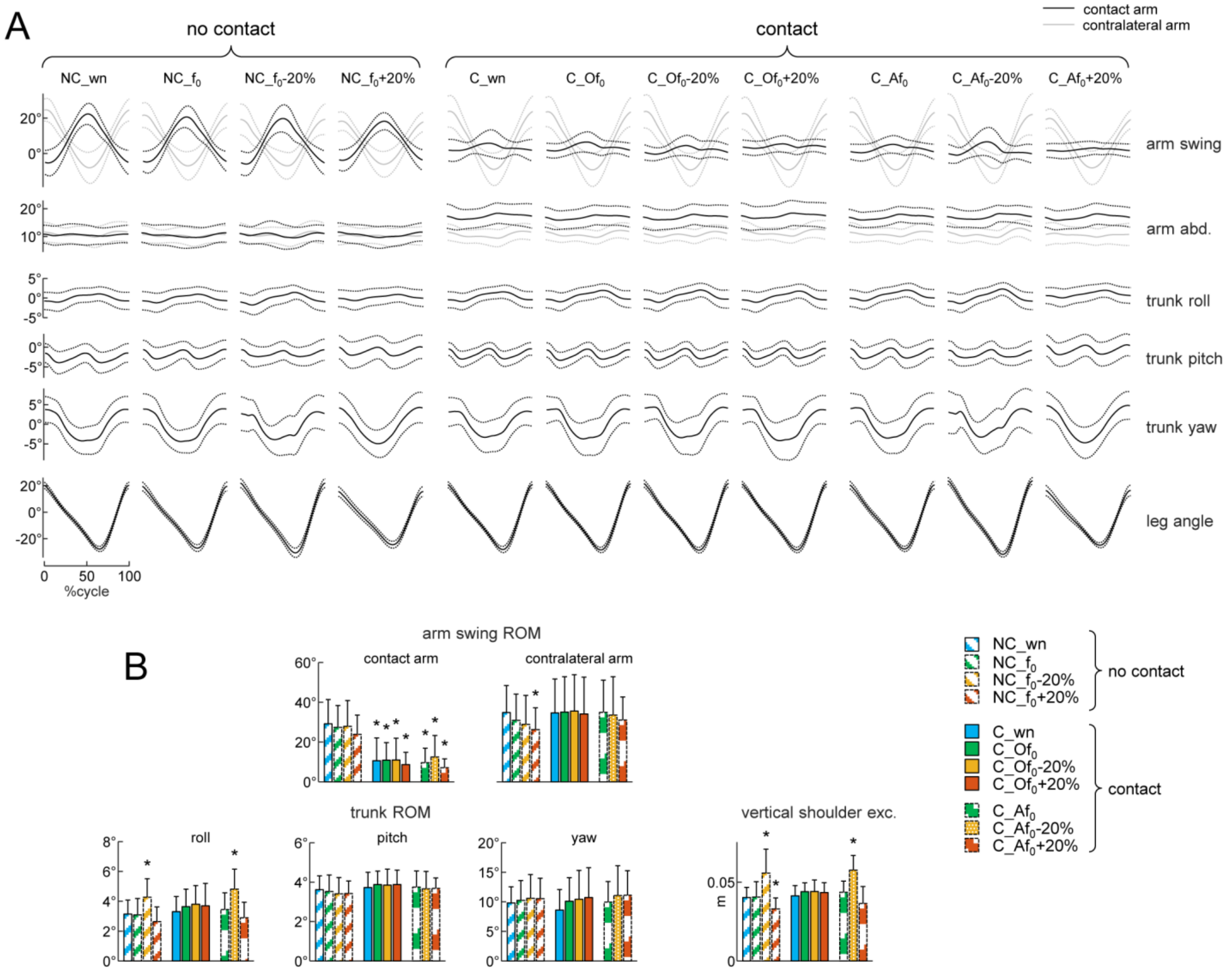

Supplementary Figure 1. Gait kinematics for all conditions. A: Ensemble averaged (mean±SD, across subjects) kinematic patterns over different conditions (see Table 2 for the abbreviations). *From top to bottom*: arm swing and abduction angles (black curves refer to the contact arm while light gray curves refer to the contralateral arm), roll, pitch and yaw angles of the trunk and contact side leg angle. Patterns are plotted versus normalized gait cycle of the contact side leg. B: averaged (+SD) range of motion (ROM) of the arm swing angle, ROM of the roll, pitch and yaw angles of the

trunk, vertical excursion of the contact side shoulder (SHO marker). Asterisks denote significant (Dunnett's post-hoc test $p<0.05$) differences relative to the normal (no contact) walking condition (NC_wn).

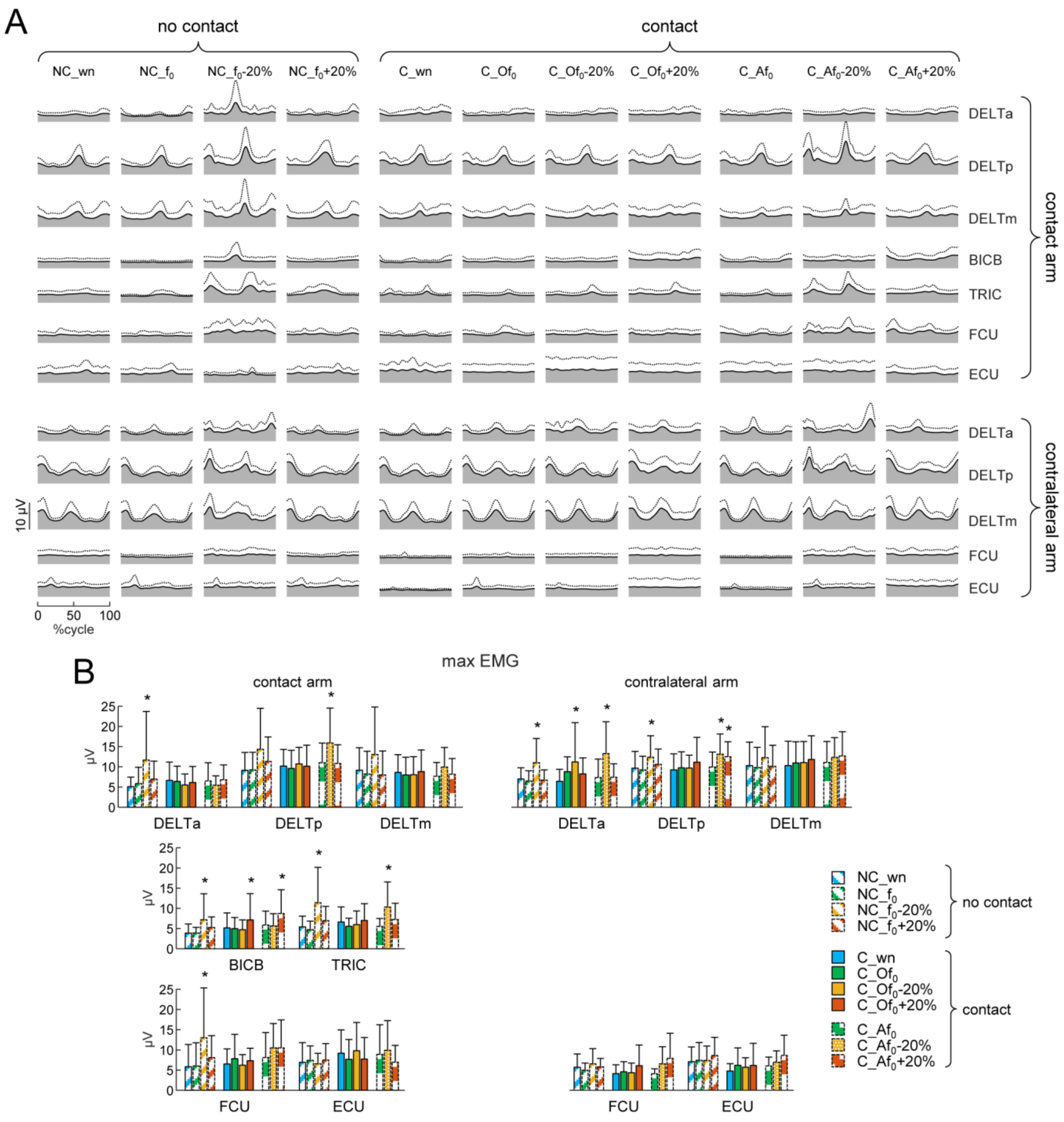

Supplementary Figure 2. Upper limb EMG patterns for all conditions. A: Ensemble averaged (mean+SD, across subjects) EMG patterns over different conditions (Table 2) for 7 muscles of the contact arm (*upper waveforms*) and 5 muscles of the contralateral arm (*lower waveforms*). Patterns are plotted versus normalized gait cycle of the contact side leg. B: maximum EMG activity (mean+SD) in the muscles of the contact (*left*) and contralateral (*right*) arm. Asterisks denote significant (Dunnett's post-hoc test $p<0.05$) differences relative to the normal (no contact) walking condition (NC_wn).
